# Interactions between cognitive and sensory load while planning and controlling complex gait adaptations in Parkinson’s disease

**DOI:** 10.1186/s12883-014-0250-8

**Published:** 2014-12-21

**Authors:** Frederico Pieruccini-Faria, Kaylena A Ehgoetz Martens, Carolina RA Silveira, Jeffery A Jones, Quincy J Almeida

**Affiliations:** Sun Life Financial Movement Disorders Research & Rehabilitation Centre, Wilfrid Laurier University, Waterloo, ON N2L 3C5 Canada; Psychology Department, Wilfrid Laurier University, Waterloo, ON N2L 3C5 Canada; Laurier Centre for Cognitive Neuroscience, Wilfrid Laurier University, Waterloo, ON N2L 3C5 Canada

**Keywords:** Parkinson’s disease, Visual feedback, Dual task, Gait with obstacle, Cognitive load

## Abstract

**Background:**

Recent research has argued that removal of relevant sensory information during the planning and control of simple, self-paced walking can result in increased demand on central processing resources in Parkinson’s disease (PD). However, little is known about more complex gait tasks that require planning of gait adaptations to cross over an obstacle in PD.

**Methods:**

In order to understand the interaction between availability of visual information relevant for self-motion and cognitive load, the current study evaluated PD participants and healthy controls while walking toward and stepping over an obstacle in three visual feedback conditions: (i) no visual restrictions; (ii) vision of the obstacle and their lower limbs while in complete darkness; (iii) vision of the obstacle only while in complete darkness; as well as two conditions including a cognitive load (with a dual task versus without a dual task). Each walk trial was divided into an early and late phase to examine changes associated with planning of step adjustments when approaching the obstacle.

**Results:**

Interactions between visual feedback and dual task conditions during the obstacle approach were not significant. Patients with PD had greater deceleration and step time variability in the late phase of the obstacle approach phase while walking in both dark conditions compared to control participants. Additionally, participants with PD had a greater number of obstacle contacts when vision of their lower limbs was not available specifically during the dual task condition. Dual task performance was worse in PD compared to healthy control participants, but notably only while walking in the dark regardless of visual feedback.

**Conclusions:**

These results suggest that reducing visual feedback while approaching an obstacle shifts processing to somatosensory feedback to guide movement which imposes a greater demand on planning resources. These results are key to fully understanding why trips and falls occur in those with PD.

## Background

It has been well documented that people with Parkinson’s disease (PD) rely more on visual feedback than healthy individuals to plan and control their movements [1-4]. Although the cause of this increased reliance on vision in PD patients is not well understood, previous studies have suggested that the reliance on visual information during goal-directed tasks may compensate for proprioceptive deficits [5-7]. Specifically, studies have demonstrated that patients with PD rely more on optic flow than healthy individuals to modulate gait parameters [7,8]. Additionally, Almeida et al. [7,9] found that patients with PD who walked towards a remembered target in a dark room had poorer estimation of the target location than healthy controls. However, when a small light-emitting diode (LED) was attached to their chest, estimation of the target location improved. These findings suggested that the visual cue for body position, aided in updating proprioceptive feedback for a motor plan. Together, these studies suggest that patients with PD are more dependent on visual feedback to update their sense of self-motion and body position compared to healthy control participants during gait. This dependence on vision may also be important for estimating the distance between their body and targets/obstacles that they have planned to negotiate in their environment.

The importance of visual feedback for perception of self-motion in highly demanding tasks, as well as for compensatory stepping right after a postural perturbation, has recently been explored. Vitorio et al. [10] recently showed decreased rates of success (more obstacle contacts) when optic flow was disrupted by strobe lighting. This study suggested that visual feedback of self-motion may be important for accurate planning (decreasing accidental obstacle contacts) for obstacle crossing, although measures of gait control during the obstacle approach were not evaluated. It is also important to note that strobe lighting might also affect participants’ perception of the obstacle’s spatial location, as well as visual feedback of the lower limbs needed for accurate clearance over an obstacle. Jacobs and Horak [11] showed that visual feedback (of the lower limbs) improves accuracy of step placement in PD patients when they are asked to step on a target during a postural task. Thus, while visual feedback has been argued to contribute to successful stepping adjustments, there have been no direct tests of the relative contribution of visual feedback on perception of self-motion, or accuracy of lower limb positioning, during complex gait tasks that involve obstacle clearance in PD. Additionally little is known about the influence of reduced visual feedback on gait control in individuals with PD when the demand for planning resources increases (i.e. walking toward an obstacle).

It is also important to consider how directing attention to relevant sensory feedback (stripes on the floor, somatosensory cues, timing cues) while walking, not only improves gait control, but is also argued to decrease processing demands required to control gait in PD [12-14]. Distorted signals from sensorimotor processing overload cognitive processing in individuals with PD [15]. Thus, sensorimotor processing affects cognitive resources in individuals with PD, especially when patients cannot use external feedback to guide their movements. The availability of relevant sensory cues are thought to help patients with PD direct their attention to key elements of locomotion, thus automating gait control in a fashion that allows individuals with PD to compensate for faulty internal modulation of steps. Greater processing demands and decreased automaticity when walking is often reflected in decreased velocity and increased step-to-step variability [16-18]. Although the relationship between sensory and cognitive load for gait control is relatively well understood [12,14], little is known about the interaction between visual feedback of self-motion and cognitive load during more complex gait tasks where planning and control are necessary to step over an obstacle.

Previous research has shown that a cognitive dual task does not affect the planning and control of step modifications to avoid an obstacle in patients with PD who have mild gait impairment [19]. It was observed that gait control in individuals with PD and healthy control participants were similarly affected by increased cognitive load during obstacle approach (where individuals plan foot clearances) and crossing (where individuals execute their motor plan). However patients in this study were tested in conditions that did not impose visual restrictions (i.e., a typically well-lit room). Therefore, it is still unknown whether reducing the availability of visual feedback for perception of self-motion and lower limb positioning (e.g., walking in a dark room towards a visible obstacle) might affect the planning resources available.

The first objective of this study was to investigate whether the impact of a dual task on gait (during obstacle approach and crossing) is amplified as visual feedback of self-motion is reduced in PD. Since planning demands may increase as participants approach an obstacle [20,21], we split the approach phase into early (far from the obstacle) and late phases (close to the obstacle). Thus, the secondary aim of this study was to evaluate whether a dual task interferes with gait, more so, in the late compared to the early phase in the reduced visual feedback conditions. It was predicted that during dual task conditions combined with decreased visual feedback about self-motion, PD patients would demonstrate slower gait velocity, and higher step-to-step variability than healthy control participants, especially as participants walked closer to the obstacle. These gait changes might indicate that visual feedback influences planning resources necessary for complex gait tasks in PD. It was also expected that there would be a greater number of obstacle contacts when visual feedback was reduced in combination with the addition of cognitive dual task. Furthermore, if reduced visual feedback results in an increased consumption of planning resources (to control gait appropriately), then we should expect that dual task performance will be worse in PD when the least amount of visual feedback is available, since this may overload planning resources.

## Methods

### Participants

Eighteen people with PD and fifteen healthy controls (HC) were recruited for the current study. There were three patients in the Hohen & Yahr stage 1.5; Seven patients in the Hohen & Yahr stage 2; Three patients in the Hoehn & Yahr stage 2.5; Five patients in the Hoehn & Yahr stage 3. All patients with PD were tested while “on” their regular anti-Parkinson’s medication. PD patients were excluded from the sample if they could not independently walk, had musculoskeletal problems, uncorrected visual problems, dementia, or other neurological or cardiac diseases. Patients with PD and HC were matched by age, height, and general cognitive status [assessed by Mini-Mental 3MS [22]] (see Table 1). The study was approved by the research ethics board at Wilfrid Laurier University, and written informed consent was obtained from all subjects prior to the experiment according to the Declaration of Helsinki.

**Table 1:** **Demographics of groups (means and standard errors)**

**GROUP**	**AGE**	**HEIGHT(cm)**	**UPDRS III**	**3MS**	**DSPAN**	**TMT-B(s)**	**TMT B-A(s)**
PD(n = 18;4F)	71.5(±7)	1.74(±4)	25.0(±6)	98.1(±3)	16.3(±3)	134.7(±14)**	94(±12)*
HC(n = 15;9F)	69.5(±6)	1.71(±5)	na	97.6(±2)	15.8(±3)	70(±15)	40(±14)

Asterisks indicate differences between groups *p < 0.05; **p < 0.01; F = females in each group; na = not available; 3MS = Mini mental 3MS; DSPAN = digit span; TMT = trail making test part B, subtraction B-A.

All comparisons were run using ANOVAs *one way* for each item in the table (except UPDRS III scores).

### Obstacle and data collection

#### Obstacle and capture area

**Figure 1:**
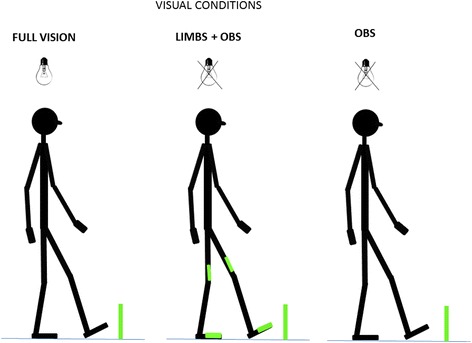
**Depiction of visual feedback conditions.** Bulbs with black cross indicate when the room was completely dark. Obstacle was visible in all conditions. Visual feedback restrictions- Full vision: no visual restriction; Limb + Obs: Obstacle and limbs visible in the dark; Obs: Only obstacle was visible in the dark.

In all trials, participants walked at a comfortable pace on a runway (gray carpet) and stepped over an obstacle. The obstacle was a bar made of white foam covered with thick white paper (70 cm width × 4 cm height × 1.5 cm depth; weight = 50 g) and supported by two lateral plastic poles (30 cm in height). The bar of the obstacle was horizontally and set at 15% of the participant’s height (~25 cm). The obstacle was positioned ~6.5 m from the starting point. The whole obstacle structure was covered with glow-in-the-dark tape. The same tape (12 cm length × 3 cm width × 0.7 mm depth) was attached along the length of the participant’s feet (aligned with the toe tips) and thighs (just above the knees) using Velcro® (Figure 1). These illuminated strips were used to provide visual information regarding the position of the participant’s knees and the anterior portion of their feet, as well as the location and height of the obstacle in the room.

#### Data recording and analysis

Participants’ movements were tracked by seven synchronized Optotrak® cameras (Northern Digital, NDI, Waterloo, Ontario): three lateral cameras on each side of the runway (vertically oriented) and one central camera (vertically oriented) 2.5 m away from the end of the runway. These cameras tracked the entire runway (~10 m). Active IREDs (infrared light emitting diodes) were fixed to the following anatomical regions: midpoint between the iliac crests (defined by the umbilicus), lateral malleolus, and 5^th^ metatarsals. Heel contacts and toe offs were visually defined using a validated method [23]. The heel contact and toe off kinematics were used to calculate gait variables during the approach and crossing phases. All kinematic data were filtered using a 2^nd^ order Butterworth filter with a cut-off frequency of 6 Hz using a dual-pass filter with zero lag delay. Kinematic variables were calculated using an algorithm created in Matlab 7.0 (The Maths Works Inc.), RRID:nlx_153890.

### Dual task

#### Cognitive task (dual task)

During this protocol participants performed the gait task protocol with the addition of a secondary task (cognitive task). The cognitive task involved attending to and counting a series of spoken digits while walking. Previous research employing dual task paradigms have involved secondary tasks which required a motor component [12-14,24-26], thus making it difficult to know if the results of these previous studies are truly due to a central overload problem, or a motor overload specifically. Therefore, the current task was chosen to avoid the confound of the secondary task having a motor component. The secondary task employed in the current study was a modified version of the protocol described in Pieruccini-Faria, Jones & Almeida [19]. In this previous research, participants were required to count two numbers that they heard from the audio track, while walking. Thus the current study eliminated the possibility that the secondary task caused motor interference (motor output overload) on the gait task. Participants were instructed to silently count the number of times they heard two different digits (assigned by the experimenter at the beginning of each trial) spoken by a female voice on an audio track. Participants heard numbers ranging from 1 to 9. The order of presentation of each digit on the audio track was randomized across the trials. During each trial the auditory inter-stimulus interval varied randomly from 100–1000 ms to prevent gait synchronization. Each stimulus (digit) presentation last 500 ms. Participants were instructed to initiate walking at the moment they heard the first digit. The audio track played for 12 s. Participants were also instructed to count until the audio track finished playing, even if they had already finished the walking task. Participants were asked to equally prioritize the gait and the digit counting task. The volume of the loudspeakers was adjusted so that participants could comfortably hear the digits at the start and end position of the walkway. At the end of each trial, participants reported the number of times they heard the target digits. Feedback about their performance was not provided. In addition to the dual task protocol, a baseline condition (BL) involving participants sitting on a chair (without visual restrictions) monitoring the digits on the audio track was also conducted. Performance on the digit counting task was calculated using the formula:

$$ Performance=\kern0.5em \left| Participan{t}^{\mathit{\hbox{'}}}s\  answer- Target\  answer\right| $$

### Visual feedback

#### Visual feedback manipulations

The experiment occurred inside a room isolated from natural light. Participants confirmed that they could not see their body or any other object when the lights were turned off. Three feedback manipulations were employed: 1) **Full vision:** the room was illuminated so that the obstacle, the environment around the obstacle, and the participants’ limbs were fully visible; 2) **Limb + Obs** condition: the room light was off, but participants could see the position of their lower limbs and the obstacle using luminescent stripes; 3) **Obs** condition: the room was dark and only the obstacle was visible. This condition was used to diminish visual feedback of self-motion and to eliminate visual feedback regarding lower limb movements. Participants completed 1 practice trial per condition (allowing us to confirm comprehension as well as the participants walking speed for normalization of the time that the dual task was made available), and then 3 trials in each visual condition with and without performing the dual task resulting in a total of 18 trials. Trials were randomized for each participant.

### Experimental protocol

#### Clinical and cognitive assessments

Motor symptom severity was assessed using the UPDRS-III (motor section) [27]. Any cognitive status declines were assessed using the Mini-mental 3MS exam [22]. Executive function related to attentional set-shifting and/or cognitive flexibility was assessed using the Trail Making Test, part A and B [28]. Participants were instructed to perform this test as fast and as accurately as they could. The motor component of the test was calculated by subtracting part A from part B. This test is considered a good predictor of cognitive flexibility, motor planning resources and mobility in patients with PD [29]. The digit span test (forward and backward) [30] was administered in order to quantify the working memory/attentional status of our participants. These tests were used to characterize the cognitive status of all participants.

#### Gait task protocol

Each participant completed a minimum of eight steps prior to stepping over the obstacle. This procedure was adopted to ensure that the time it took for each participant to perform the dual task was similar. After each trial the starting position was adjusted 30 cm forward or backwards so that participants could not predict which leg they would step over the obstacle with.

### Data analyses and statistics

#### Gait analysis

##### Gait Parameters during approaching phase

The data capture area permitted the analysis of the last eight steps prior to obstacle crossing. However, to remove gait characteristics associated with gait initiation, only the last 6 steps prior to the obstacle were analysed. These six steps were divided into two phases, an early phase and a late phase, each containing 3 steps. The speed of gait was calculated as the average of the step velocity of the three steps in each phase. Step-to-step time and length variability were calculated using the coefficient of variation (CV) of steps in each phase ((Standard deviation/Mean)*100).

### Obstacle crossing parameters

Lead toe clearance was calculated by subtracting the vertical position of the 5^th^ metatarsal marker on each foot from the obstacle’s height, at the frame or instant when the foot was directly over top of the obstacle (i.e., the crossing point). Trail horizontal distance before the obstacle and lead horizontal distance beyond the obstacle were captured as horizontal distances between the foot and the obstacle, subtracting the position of the marker on the 5^th^ metatarsal of each foot from the obstacle position in the sagittal plane (Figure 1).

#### Statistical analyses

In order to investigate the motor planning difficulties, step-velocity and step-variability were analysed using a two-way mixed repeated measures analysis of variance (RM ANOVA) with group (PD, Healthy controls (HC)) as a between-subjects factor on gait velocity, step-to-step time variability and step-to-step length variability [Conditions: visual feedback (3) × task (2) × phases (2)]. In order to investigate how conditions influenced foot clearance, another two-way mixed RM ANOVA with group (PD, HC) as a between-subjects factor [Conditions: visual feedback (3) × task (2)] was used to observe the interactions between task and visual feedback on trail-limb horizontal distance before obstacle, lead-limb toe clearance, lead horizontal distance beyond obstacle and their variability (standard deviation of these distances). Tukey-HSD post hocs were applied when appropriate. The motor planning errors (obstacle contacts) were analyzed using non-parametric tests. Kruskal-Wallis and the Wilcoxon test were used to compare the rate of success of obstacle crossing. Differences were accepted when p values were ≤0.050. All statistical analyses were run in STATISTICA 8.0.

## Results

### Baseline gait measures

Overall, the PD group showed gait characteristics that are typically observed in patients with PD: shorter step length (PD: 54.0 cm ±1.9, HC: 64.4 cm ± 2.1; F_1, 31_ = 12.72, p = 0.001) and slower gait speed (PD: 99.1 ± 4.0 cm/s, HC: 126.2 cm/s ±4.4; F_1, 31_ = 15.41, p < 0.001).

### Gait during obstacle approach

#### Gait velocity

**Figure 2:**
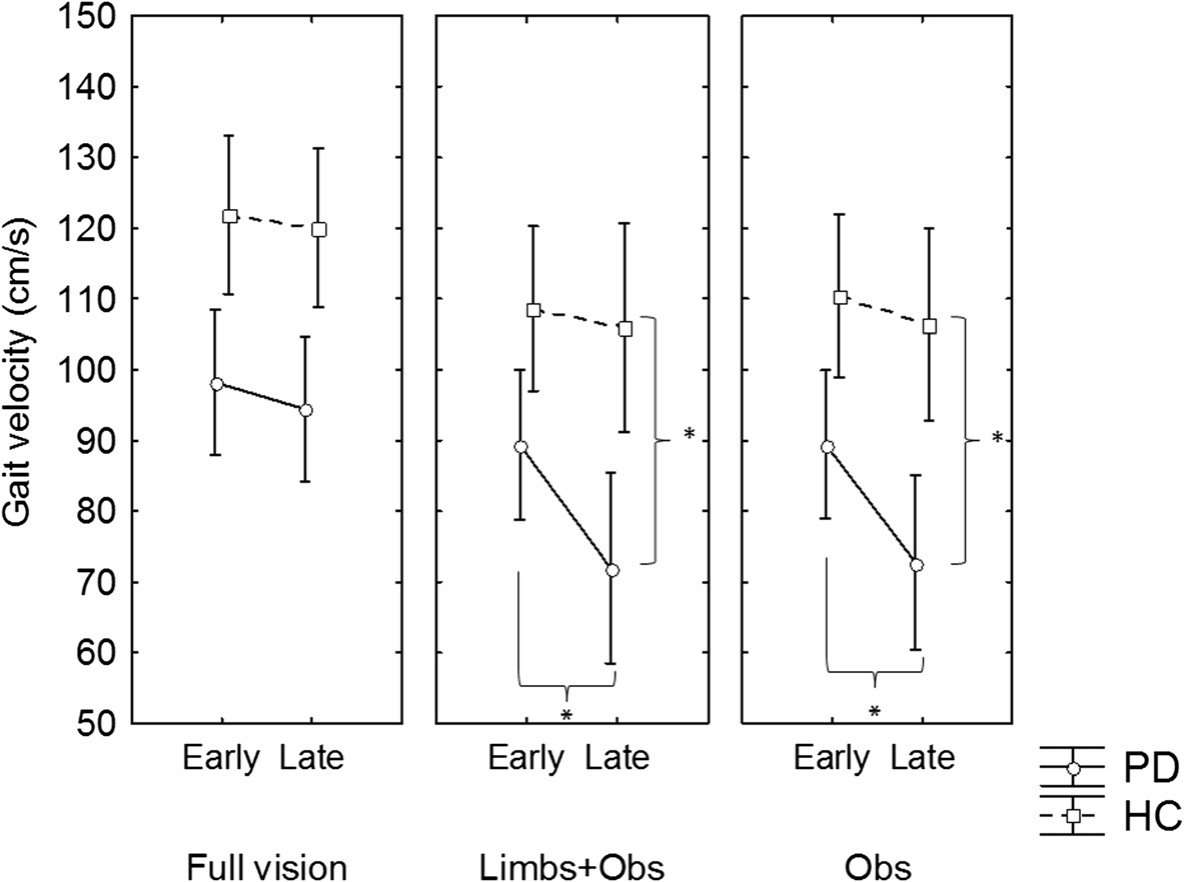
**Significant interactions between Phase x Vision x Group.** PD patients had greater magnitude of deceleration when walking in the darkness compared to healthy control participants. *p < 0.05.

The hypothesized interactions between group, visual conditions, dual task and phase did not reach statistical significance. However, the results for gait velocity during obstacle approach showed significant main effects of group (PD patients were slower than the healthy controls) (F_1,31_ = 16.67; p = 0.001), phases (Participants were slower in the late phase compared to early phase) (F_1,31_ = 67.76; p < 0.001) and task (both groups were slower when performing a dual task). A main effect of visual feedback (F_1,62_ = 61.82; p < 0.001) was also found and post hoc tests revealed that participants in general were slower in the Limb + Obs and Obs compared to Full vision condition. A three-way interaction between Group x visual feedback x phase for gait velocity (F_2,62_ = 4.05; p = 0.02) revealed that PD patients reduced their walking speed (i.e., greater deceleration in the late phase compared to early phase) more than healthy control participants during their approach of the obstacle when the room was dark (Obs and Limb + Obs) (Figure 2).

#### Step time variability

**Figure 3:**
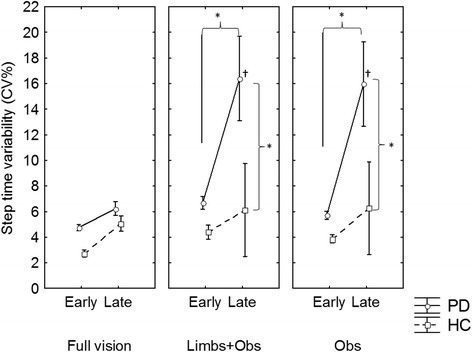
**PD patients had an increase in step time variability when approaching the obstacle only in the dark.** *p < 0.05; † different from late phase in the full vision condition.

The hypothesized interactions between group, visual conditions, dual task and phase did not reach statistical significance. Main effects of group (F_1,31_ = 5.39; p = 0.021) (PD were more variable than HC), and phase (F_1,31_ = 14.14; p = 0.001) (participants were more variable in the late phase) were also found for step time variability (see Table 2). A main effect of visual feedback (F_1,62_ = 9.52; p < 0.001) was also found and post hoc tests revealed that all participants were more variable in the dark conditions compared to full vision. Additionally, a three-way interaction between group, visual feedback, and phase (F_2,62_ = 4.14; p = 0.02) was identified for step time variability. Post hoc revealed that in the Obs and Limb + Obs conditions PD patients increased step time variability more so in the late phase (compared to early phase) than healthy control participants (Figure 3), with these group differences apparent in only the late phase of their approach.

**Table 2:** **Mean and standard errors (in brackets) of gait parameters during obstacle approach in each phase**

**Groups**	**PHASE**	**Task**	**Gait velocity(cm/s)**	**Step time variability(%CV)**	**Step length variability(%CV)**
PD	Early	No dual task	99.5(±12.6)	5.08(±0.7)	7.29(±3.4)
	Early	Dual task	87.8(±12.9)	6.33(±1.1)	5.79(±2.1)
	Late	No dual task	85.8(±15.3)	12.27(±6.2)	10.74(±3.4)
	Late	Dual task	76.2(±13.1)	13.45(±8.1)	11.13(±3.1)
HC	Early	No dual task	120.3(±13.8)	3.59(±0.8)	2.82(±3.7)
	Early	Dual task	106.9(±14.1)	3.73(±1.2)	2.92(±2.3)
	Late	No dual task	115.8(±16.8)	5.52(±6.8)	8.82(±3.7)
	Late	Dual task	105.7(±14.4)	6.10(±8.9)	8.07(±3.4)
Effects		Group	P < 0.001	P < 0.05	P < 0.001
		Task	P < 0.001	NS	NS
		Phase	P < 0.05	P < 0.01	P < 0.001
		Group x task	NS	NS	NS
		Group x phase	NS	NS	NS
		Group x task x phase	NS	NS	NS

NS – not significant.

Visual conditions are collapsed in each task condition.

#### Step length variability

The hypothesized interactions between group, visual conditions, dual task and phase did not reach statistical significance. Main effects of group (F_1,31_ = 10.07; p = 0.003) (PD patients were more variable than healthy controls), and phase (F_1,31_ = 32.52; p < 0.001) (all participants were more variable in the late phase) were identified for step-length variability; however no interactions were significant. A main effect of visual feedback (F_2,62_ = 4.10; p = 0.021) was found, and post hoc tests revealed that all participants were more variable in the Obs condition compared to Limbs + Obs but not compared to Full vision.

#### Foot clearances

Groups had similar foot-to-obstacle distances during obstacle crossing. There were no interactions between group, visual conditions, or task. We found a significant interaction between visual feedback and task (F_2,62_ = 5.62; p = 0.001) for toe clearances. Post hoc revealed that lead toe clearances during Limb + Obs and Obs were larger compared to full vision, but it was shorter when performing the dual task only during Limb + Obs and Obs. A significant main effect of visual condition was found for trail horizontal distance before obstacle crossing (F_2,62_ = 4.17; p = 0.004). Post hoc revealed that all groups placed their feet farther from the obstacle during Limb + Obs and Obs conditions (see Table 2). Significant main effects of visual feedback (F_2, 62_ = 59.34, p < 0.001) and task (F_1, 31_ = 17.94, p < 0.001) were also identified for lead horizontal distances beyond obstacle. Post hoc revealed that during Obs and Limb + Obs and dual task conditions, all participants had shorter lead horizontal distances beyond obstacle (see Table 3), compared to during full vision.

**Table 3:** **Mean and standard errors of crossing variables (foot-to-obstacle distances) and its variability (standard deviation)**

	**Conditions**	**Trail horizontal distance before obstacle(cm)**	**Lead toe clearance(cm)**	**Lead horizontal distance beyond obstacle(cm)**	**Trail horizontal distance before obstacle variability(cm)**	**Lead toe clearance variability(cm)**	**Lead horizontal distance beyond obstacle variability(cm)**	**Crossing velocity (cm/s)**
PD	Obs	30.44(±3.8)	23.86(±2.9)	35.26(±3.3)	3.64(±0.5)	1.96(±0.2)	3.55(±0.4)	440.0(±88.6)
	Obs + DT	30.41(±3.0)	23.23(±2.3)	32.72(±2.8)	4.09(±0.4)	1.92(±0.2)	3.80(±0.4)	381.1(±75.7)
	Limb + Obs	30.65(±3.4)	24.09(±2.5)	35.09(±2.8)	4.39(±0.5)	2.74(±0.3)	3.49(±0.4)	418.7(±86.7)
	Limb + Obs + DT	29.10(±2.7)	22.89(±2.3)	34.13(±2.3)	2.39(±0.5)	1.83(±0.2)	2.25(±0.3)	393.4(±73.9)
	Full vision	28.29(±3.5)	19.06(±2.3)	41.71(±2.7)	4.02(±0.4)	1.65(±0.1)	3.09(±0.4)	605.3(±79.3)
	Full vision + DT	27.29(±3.4)	18.90(±2.3)	39.63(±2.8)	3.69(±0.5)	1.92(±0.3)	3.51(±0.4)	551.4(±75.2)
HC	Obs	29.30(±4.2)	27.45(±3.2)	38.12(±3.6)	3.59(±0.5)	1.91(±0.3)	3.17(±0.5)	550.7(±97.1)
	Obs + DT	29.75(±3.3)	26.14(±2.5)	36.19(±3.0)	3.77(±0.5)	2.25(±0.2)	2.63(±0.5)	556.1(±73.0)
	Limb + Obs	29.74(±3.8)	26.02(±2.7)	37.25(±3.7)	4.07(±0.6)	1.80(±0.3)	4.49(±0.5)	582.5(±95.0)
	Limb + Obs + DT	29.42(±3.0)	25.24(±2.5)	36.75(±2.5)	3.87(±0.6)	2.48(±0.3)	3.28(±0.4)	560.5(±83.0)
	Full vision	27.67(±3.8)	21.59(±2.5)	45.36(±3.0)	3.73(±0.4)	1.68(±0.2)	3.65(±0.5)	747.6(±86.9)
	Full vision + DT	26.53(±3.8)	20.33(±2.6)	42.49(±3.1)	3.40(±0.6)	2.25(±0.3)	3.12(±0.5)	664.7(±82.4)
Effects	Group	NS	NS	NS	NS	NS	NS	P = 0.02
	Vision	P < 0.001	P < 0.001	P < 0.001	NS	NS	NS	P < 0.001
	Task	NS	P < 0.001	P < 0.001	NS	NS	NS	P = 0.01
	Group x vision	NS	NS	NS	NS	NS	NS	NS
	Group x Task	NS	NS	NS	NS	NS	NS	NS
	Vision x Task	NS	P < 0.001	NS	NS	NS	NS	NS
	Group x vision x task	NS	NS	NS	NS	NS	NS	NS

#### Variability of the foot clearances

The variability of foot clearances was not influenced by conditions and was similar between groups (Table 3).

#### Crossing velocity

Individuals with PD crossed the obstacle slower than healthy controls in all conditions (F_1, 31_ = 5.29, p = 0.02). Participants crossed the obstacle slower when performing the dual task (F_2, 62_ = 70.78, p < 0.001). Participants also crossed the obstacle slower when walking in the dark with or without glow-in-the-dark tape attached to their lower limbs (F_1, 31_ = 7.5, p = 0.01). There were no significant interactions.

#### Obstacle contacts during obstacle crossing

**Figure 4:**
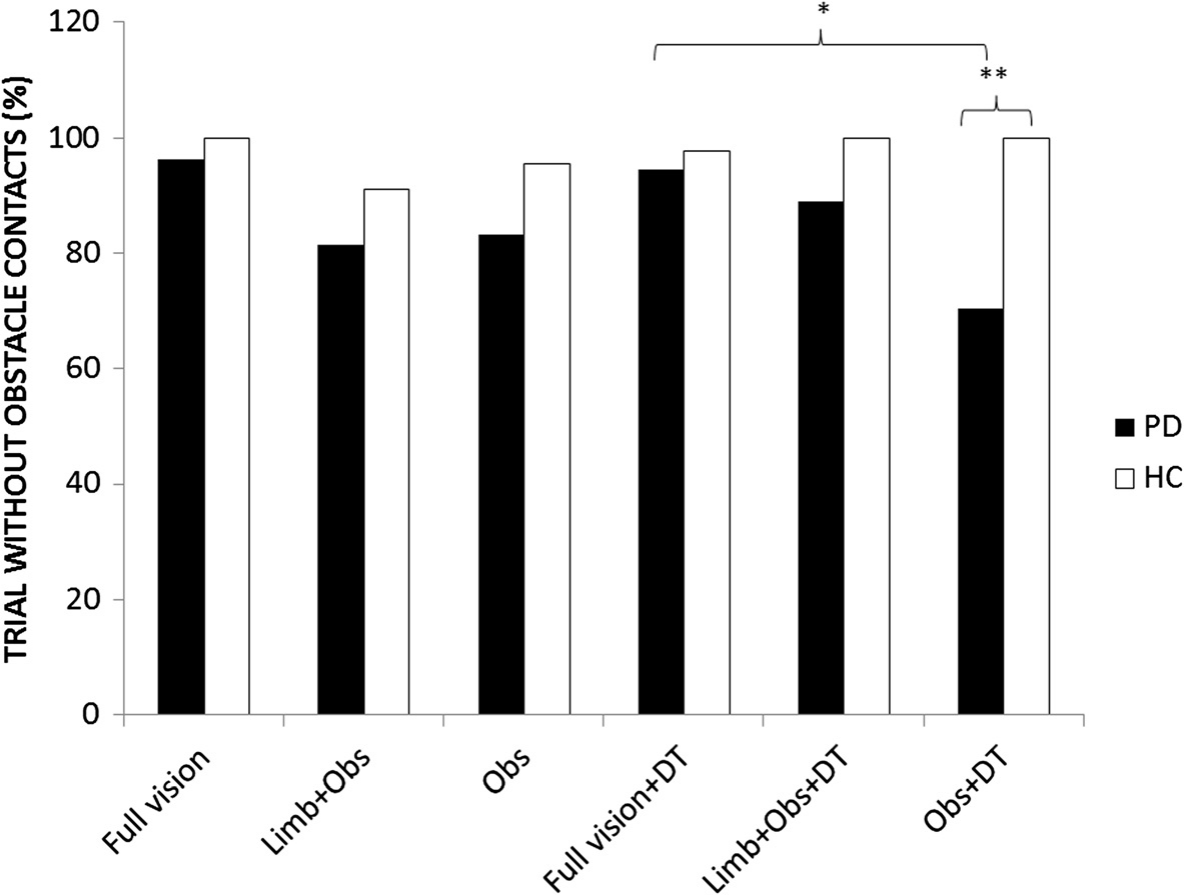
**Bars represent the percentage of successful crossings in each condition for each group.** *p < 0.05; **p < 0.01.

Since the rate of success during obstacle crossing was not normally distributed, non-parametric tests were used to compare groups in each condition. The interaction between group, visual condition and dual task was found when running non parametric tests for the percentage of obstacle contacts. A Kruskal-Wallis ANOVA revealed that PD patients had lower rates of success compared to healthy controls participants in the Obs + DT condition (*χ*^2^ = 9.71; df = 1, p =0.002). Wilcoxon tests revealed a lower rate of success during obstacle crossing (more obstacle contacts) amongst PD patients during the Obs + DT condition compared to Full vision + DT(p = 0.012); (Figure 4).

#### Digit monitoring performance

Performance on the dual task was not normally distributed; hence non-parametric tests were used to compare groups in each condition. A Kruskal-Wallis ANOVA test for independent samples revealed that patients with PD were less accurate than healthy control participants when monitoring digits in the Obs condition (*χ*^2^ = 4.1; df = 1, p = 0.04). Paired comparisons using Wilcoxon tests for dependent samples revealed that PD group tended to be less accurate in the Obs (p = 0.07) and less accurate in the Full vision condition (p = 0.02) compared to baseline. Healthy control participants tended to have greater counting errors during full vision compared to baseline (p = 0.058) (see Table 4).

**Table 4:** **Accuracy of the answers (answer error mean) of each group for each visual condition**

	**BL**	**Full vision**	**Limb+Obs**	**Obs**
PD	1.75(±0.29)	2.53(±0.30)^a^	2.29(±0.43)	2.37(±0.29)^b^
HC	1.51(±0.31)	2.00(±0.33)	1.42(±0.47)	1.64(±0.32)

Legend - BL = base line condition (performing the cognitive task sitting on a chair); ^a^different from baseline p < 0.05; ^b^difference between groups p < 0.05.

Greater numbers represent worse performance. A zero score would represent an exact answer.

## Discussion

The overall objective of this study was to investigate whether the impact of a dual task on gait (during obstacle approach and crossing) is amplified as visual feedback of self-motion is reduced in PD. While approaching an obstacle, utilization of planning resources increases as one gets closer to the obstacle. Thus, the secondary aim of this study was to evaluate whether a dual task interferes with gait, more so, in the late compared to the early phase in the reduced visual feedback conditions. It was found that when visual feedback about self-motion was reduced, individuals with PD had greater number of errors in the dual task compared to healthy control participants. Additionally, individuals with PD had a greater number of obstacle contacts specifically while walking with reduced visual feedback of self-motion and with the dual task compared to healthy control participants. Yet, the dual task influenced gait similarly in individuals with PD and healthy control participants, regardless of visual feedback manipulations. Furthermore, the dual task did not affect gait differently in the early and late phases. In summary, the dual task did not interfere with gait in either group, however, the increased number of obstacle contacts by individuals with PD, in the darkness (Obs), might suggest that the dual task interfered with planning during the late phase, when gait was most affected by reduced visual feedback; or shared resources in those with PD reducing their ability to process sensory feedback during obstacle crossing.

In this study, individuals with PD had worse performance on the cognitive task (i.e. number counting) while walking in the dark specifically when only the obstacle was visible (Obs condition) compared to healthy control participants. It is important to note that at baseline condition (i.e. when counting numbers seated) participants with PD performed similar to healthy participants (see Table 4), highlighting that deficits in PD are specifically associated with reduction of visual feedback. This result suggests that individuals with PD may have been prioritizing the gait task when walking in the dark with reduced self-motion feedback. Prioritizing gait might be a strategy that individuals with PD employ, to allocate more resources (e.g. attention) to the processing of sensory information when critical pieces of visual information are not available. This notion that cognitive resources compensate poor sensorimotor integration, has been supported by previous research that has shown that when visual feedback of self-motion is not available, elderly people allocate more attentional resources to their postural control [31,32]. Similar results are found in gait when proprioceptive feedback is reduced by peripheral neurological diseases [33,34]. Although dual task performance suffered, prioritization of gait likely allowed those with PD to control gait during the approach, in a similar fashion to healthy control participants. Additionally, our results are in line with recent theory, supporting the notion that individuals with PD operate in an attention-controlled mode due to an abnormal sensorimotor processing within basal ganglia loops [15]. Hence, PD patients might be using more central resources to overcome distorted sensorimotor signals when visual feedback of self-motion is not fully available to achieve gait control.

Previous research has shown that sensory cues reduce the interference of a secondary motor task by reducing the demand on central resources [12,14]. In the current study, neither adding (i.e. Limb + Obs) nor reducing (i.e. Obs) visual feedback influenced the interference of the cognitive task on gait. This was contrary to our hypothesis and might be explained by the nature of the secondary task (e.g. carrying a tray with cups while walking) employed in these other studies. It might be the case that in previous studies, providing sensory cues may have made one of the motor tasks more automatic, however this did not directly evaluate whether sensory cues influence cognitive resources available. It is important to note that in the current study, the secondary task was purely cognitive, with the intention of understanding the demand of cognitive processing irrespective of motor interference. Therefore, based on the findings from this study, it appears that cognitive resources are used to compensate for the reduction of sensory feedback, to lessen the interference of the cognitive task and more successfully control gait in a task that involves increased postural threat.

Although foot clearance variables were not different between groups, we found that individuals with PD contacted the obstacle more frequently than healthy controls, specifically when PD participants walked with reduced self-motion visual feedback (Obs) and a dual task (Figure 4). One possible reason for this discrepancy may be that our measure of toe clearance was based on distance from 5^th^ metatarsal to obstacle, but did not take into account other parts of the foot (such as heel or shank of leg) that could have contacted the obstacle. This discrepancy has also been reported in a previous study that employed this same measure [10], and might explain why toe clearances were similar between groups while obstacle contacts were greater in those with PD. This result highlights how reduced self-motion visual feedback taxes central resources in PD. As a result of shared resources, motor planning may have been affected, resulting in greater number of obstacle contacts. Alternatively, shared central resources might impair one’s ability to effectively process sensory feedback [34,35] or update sensory feedback into a motor plan [9] during obstacle crossing. Evidence from this study showed that providing additional visual feedback about lower limb position (i.e. Limb + Obs) minimized obstacle contacts during dual-task conditions (participants performed similar to healthy controls and their own performance in the full vision condition). This finding suggests that visual feedback of lower limb position compensates for proprioceptive impairment in PD as suggested by previous research [36-37]. Importantly, when visual feedback is removed (i.e. in complete darkness) individuals with PD may allocate more attentional resources to the sampling of proprioceptive feedback, in order to compensate for the limited sensory feedback available. Increased number of errors with the dual task supports the notion that PD participants allocated more attentional resources to proprioceptive feedback while walking. Yet, it is perhaps peculiar that gait toward the obstacle did not improve. Given that the current gait task was very goal-oriented with the focus on not tripping over the obstacle, it is likely that attentional resources were primarily dedicated to planning how to clear the obstacle rather than the gait characteristics during the approach. The fact that obstacle contacts were greater in the dark (Obs) compared to (Limbs + Obs) supports this notion since more resources were allocated to obstacle clearance (perhaps with vision of foot and obstacle to affirm safe clearance), while gait during the approach seems to indicated that PD participants were unable to fully compensate for proprioceptive deficits [6,36]. This finding can be further evaluated by examining the role of sensory feedback while approaching the obstacle.

A confirmation of the key role of sensory feedback especially in the late phase was demonstrated by significant deceleration (see Figure 1) and increased step time variability (see Figure 2) specifically in participants with PD in the late phase (but not healthy participants). This change in behaviour was only evident when individuals with PD were required to walk in the dark with reduced visual feedback (both Obs and Limb + Obs). Gait deceleration might reflect a strategy used by individuals with PD to provide more time to process incoming sensory information, as suggested by previous studies in elderly people [38,39]. Additionally, some researchers have suggested that increased step time variability represents difficulties to integrate sensory feedback to achieve timing control [40]. Step time variability is also linked to less automatic gait control [17], likely caused by greater dedication of resources to monitor sensorimotor processes. Therefore, it is important to consider that the late phase demands greater sensory integration to control movement just prior to crossing the obstacle, which may be why these differences are not seen in the early phase. Previous research has shown that visual feedback of body position improves gait control in PD while walking in the dark [9]. Although the current study did not find that visual feedback of body position improved gait in the late phase, it was able to prevent obstacle contacts during the crossing phase when the cognitive load was increased. It is possible that visual attention of patients was mostly concentrated on obstacle and 2–3 steps ahead, as suggested by previous studies with healthy adults [41,42]. Thus characteristics of visual feedback utilization to avoid an obstacle may have impeded patients to fully use visual feedback from lower limbs. This might suggest that providing feedback about body limb position may provide partial compensation for proprioceptive deficits during more demanding gait adaptations in PD.

Although we have not included individuals with freezing of gait in this study, our PD patients demonstrated gait behaviours similar to individuals who experience freezing of gait while approaching narrow doorways (e.g. abnormal gait deceleration and increased step-to-step variability) [43]. Previous studies have suggested that these severe gait behaviours may be associated with an abnormal response to action-relevant stimuli in the environment [44]. Increased salience of action-relevant stimulus (a lit doorway in the dark) can cause greater freezing than a normally lit room [45]. However, there is also evidence showing that individuals with freezing of gait demonstrate more difficulties integrating vision and proprioception during a motor task [46]. Therefore, an abnormal gait response to visual information (obstacle in the dark) and the necessity to integrate more somatosensory feedback (e.g. proprioception) into a motor plan may be an important underlying mechanism that explains more severe gait deficits such as freezing.

It is also important to acknowledge that walking in the dark could have generated anxiety among individuals with PD. Reduced visual feedback of self-motion may exacerbate balance problems in individuals with PD, which increases the chances of falling [47]. Anxiety, created by postural threats, influences obstacle crossing kinematics of older adults, such as foot clearances and crossing speed [48]. However, in current study, individuals with PD and healthy controls had similar crossing behaviours in the dark. Thus, it is unlikely that increased anxiety has contributed to the results in current study. Future studies could explore this issue further.

### Limitations

This study has some limitations that need to be acknowledged. The number of steps used to calculate step-to-step time variability is low compared to previous research [17]. However, variability between phases using the same number of steps for all groups was consistently compared. Other studies have also calculated step time variability from the same amount of steps [19,49]. Another limitation is that it was not possible to know the performance of the secondary task in each phase. It might be possible that the performance of the secondary task in each phase changed as participants approached the obstacle. Poor performance in the secondary task would also indicate that the demand for central resources (e.g. cognitive processes, attention) during obstacle approach increased.

## Conclusion

The current study sheds light on the importance of central resources for sensorimotor processing when individuals with PD are planning and controlling gait during obstacle avoidance. Visual feedback about self-motion reduces the demand on cognitive resources, however, this does not fully compensate for proprioceptive deficits in PD. Impairments in sensorimotor processing in PD could deplete planning resources which affects their ability to avoid obstacles safely. In sum, impaired gait adaptability in PD patients may result from interactions between sensory and cognitive processing. From a clinical point of view, gait therapy programs for individuals with PD should include visual feedback and cognitive load manipulations to improve their safety and gait adaptability.

## References

[1] Desmurget M, Grafton ST, Vindras P, Grea H, Turner RS (2003). Basal ganglia network mediates the control of movement amplitude. Experimental Brain Research.

[2] Desmurget M, Grafton ST, Vindras P, Grea H, Turner RS (2004). The basal ganglia network mediates the planning of movement amplitude. Eur J Neurosc.

[3] Klockgether T, Dichgans J (1994). Visual control of arm movement in Parkinson's disease. Movement Disord.

[4] Ghilardi MF, Alberoni M, Rossi M, Franceschi M, Mariani C, Fazio F (2000). Visual feedback has differential effects on reaching movements in Parkinson's and Alzheimer's disease. Brain Research.

[5] Adamovich SV, Berkinblit MB, Hening W, Sage J, Poizner H (2001). The interaction of visual and proprioceptive inputs in pointing to actual and remembered targets in Parkinson's disease. Neuroscience.

[6] Contreras-Vidal JL, Gold DR (2004). Dynamic estimation of hand position is abnormal in Parkinson's disease. Parkinsonism Relat D.

[7] Azulay JP, Mesure S, Amblard B, Blin O, Sangla I, Pouget J (1999). Visual control of locomotion in Parkinson's disease. Brain.

[8] Schubert M, Prokop T, Brocke F, Berger W (2005). Visual kinesthesia and locomotion in Parkinson's disease. Movement Disord.

[9] Almeida QJ, Frank JS, Roy EA, Jenkins ME, Spaulding S, Patla AE, Jog MS (2005). An evaluation of sensorimotor integration during locomotion toward a target in Parkinson's disease. Neuroscience.

[10] Vitorio R, Lirani-Silva E, Barbieri FA, Raile V, Stella F, Gobbi LT: **Influence of visual feedback sampling on obstacle crossing behavior in people with Parkinson's disease.** *Gait & posture*ᅟ:ᅟ 2013. 10.1016/j.gaitpost.2012.12.019 23347768

[11] Jacobs JV, Horak FB (2006). Abnormal proprioceptive-motor integration contributes to hypometric postural responses of subjects with Parkinson's disease. Neuroscience.

[12] Rochester L, Nieuwboer A, Baker K, Hetherington V, Willems AM, Chavret F, Kwakkel G, Van Wegen E, Lim I, Jones D (2007). The attentional cost of external rhythmical cues and their impact on gait in Parkinson's disease: effect of cue modality and task complexity. Journal of Neural Transmition.

[13] Baker K, Rochester L, Nieuwboer A (2007). The immediate effect of attentional, auditory, and a combined cue strategy on gait during single and dual tasks in Parkinson's disease. Archives of Physical Medicine & Rehabilitation.

[14] van Wegen E, de Goede C, Lim I, Rietberg M, Nieuwboer A, Willems A, Jones D, Rochester L, Hetherington V, Berendse H, Zijlmans J, Wolters E, Kwakkel G (2006). The effect of rhythmic somatosensory cueing on gait in patients with Parkinson's disease. Journal of the neurological sciences.

[15] Redgrave P, Rodriguez M, Smith Y, Rodriguez-Oroz MC, Lehericy S, Bergman H, Agid Y, DeLong MR, Obeso JA (2010). Goal-directed and habitual control in the basal ganglia: implications for Parkinson's disease. Nature reviews Neuroscience.

[16] Hausdorff JM, Balash J, Giladi N (2003). Effects of cognitive challenge on gait variability in patients with Parkinson's disease. Journal of Geriatrics, Psychiatry & Neurology.

[17] Yogev G, Giladi N, Peretz C, Springer S, Simon ES, Hausdorff JM (2005). Dual tasking, gait rhythmicity, and Parkinson's disease: which aspects of gait are attention demanding?. Eur J Neurosc.

[18] O'Shea S, Morris ME, Iansek R (2002). Dual task interference during gait in people with Parkinson disease: effects of motor versus cognitive secondary tasks. Physical therapy.

[19] Pieruccini-Faria F, Jones JA, Almeida QJ (2014). Motor planning in Parkinson's disease patients experiencing freezing of gait: The influence of cognitive load when approaching obstacles. Brain & Cognition.

[20] Bradshaw EJ, Sparrow WA (2001). Effects of approach velocity and foot-target characteristics on the visual regulation of step length. Hum Movement Sci.

[21] Sparrow WA, Bradshaw EJ, Lamoureux E, Tirosh O (2002). Ageing effects on the attention demands of walking. Hum Movement Sci.

[22] Teng EL, Chui HC (1987). The Modified Mini-Mental State (3MS) examination. J Clin Psychiat.

[23] O'Connor CM, Thorpe SK, O'Malley MJ, Vaughan CL (2007). Automatic detection of gait events using kinematic data. Gait & posture.

[24] Canning CG (2005). The effect of directing attention during walking under dual-task conditions in Parkinson's disease. Parkinsonism Relat Disord.

[25] Bond JM, Morris M (2000). Goal-directed secondary motor tasks: Their effects on gait in subjects with Parkinson disease. Arch Phys Med Rehab.

[26] Brauer SG, Woollacott MH, Lamont R, Clewett S, O'Sullivan J, Silburn P, Mellick GD, Morris ME (2011). Single and dual task gait training in people with Parkinson's disease: a protocol for a randomised controlled trial. BMC Neurology.

[27] Goetz CG, LeWitt PA, Weidenman M (2003). Standardized training tools for the UPDRS activities of daily living scale: newly available teaching program. Movement Disord.

[28] Fitzhugh KB, Fitzhugh LC, Reitan RM (1962). Relation of acuteness of organic brain dysfunction to Trail Making Test performances. Perceptual and Motor Skills.

[29] Xanthopoulos P, Heilman KM, Drago V, Pardalos P, Foster PS, Skidmore FM (2008). An ambulatory persistence power curve: motor planning affects ambulatory persistence in Parkinson's disease. Neuroscience letters.

[30] Blackburn HL, Benton AL (1957). Revised administration and scoring of the digit span test. Journal of consulting psychology.

[31] Meyer E, Ferguson SS, Zatorre RJ, Alivisatos B, Marrett S, Evans AC, Hakim AM (1991). Attention modulates somatosensory cerebral blood flow response to vibrotactile stimulation as measured by positron emission tomography. Annals of neurology.

[32] Teasdale N, Simoneau M (2001). Attentional demands for postural control: the effects of aging and sensory reintegration. Gait & posture.

[33] Courtemanche R, Teasdale N, Boucher P, Fleury M, Lajoie Y, Bard C (1996). Gait problems in diabetic neuropathic patients. Archives of Physical Medicine & Rehabilitation.

[34] Lajoie Y, Teasdale N, Cole JD, Burnett M, Bard C, Fleury M, Forget R, Paillard J, Lamarre Y (1996). Gait of a deafferented subject without large myelinated sensory fibers below the neck. Neurology.

[35] Pashler H (1994). Dual-task interference in simple tasks: data and theory. Psychological Bulletin.

[36] Konczak J, Li KY, Tuite PJ, Poizner H (2008). Haptic perception of object curvature in Parkinson's disease. PLoS One.

[37] Konczak J, Sciutti A, Avanzino L, Squeri V, Gori M, Masia L, Abbruzzese G, Sandini G (2012). Parkinson's disease accelerates age-related decline in haptic perception by altering somatosensory integration. Brain.

[38] Rosano C, Studenski SA, Aizenstein HJ, Boudreau RM, Longstreth WT, Newman AB (2012). Slower gait, slower information processing and smaller prefrontal area in older adults. Age & Ageing.

[39] Watson NL, Rosano C, Boudreau RM, Simonsick EM, Ferrucci L, Sutton-Tyrrell K, Hardy SE, Atkinson HH, Yaffe K, Satterfield S, Harris TB, Newman AB, Health ABC Study (2010). Executive function, memory, and gait speed decline in well-functioning older adults. The journals of gerontology Series A, Biological sciences and medical sciences.

[40] Almeida QJ, Frank JS, Roy EA, Patla AE, Jog MS (2007). Dopaminergic modulation of timing control and variability in the gait of Parkinson's disease. Movement Disord.

[41] Patla AE, Vickers JN (1997). Where and when do we look as we approach and step over an obstacle in the travel path?. Neuroreport.

[42] Mohagheghi AA, Moraes R, Patla AE (2004). The effects of distant and on-line visual information on the control of approach phase and step over an obstacle during locomotion. Experimental Brain Research.

[43] Almeida QJ, Lebold CA (2010). Freezing of gait in Parkinson's disease: a perceptual cause for a motor impairment?. J Neurol Neurosur Ps.

[44] Cowie D, Limousin P, Peters A, Day BL (2010). Insights into the neural control of locomotion from walking through doorways in Parkinson's disease. Neuropsychologia.

[45] Ehgoetz Martens KA, Pieruccini-Faria F, Almeida QJ (2013). Could sensory mechanisms be a core factor that underlies freezing of gait in Parkinson's disease?. PLoS One.

[46] Tan T, Almeida QJ, Rahimi F (2011). Proprioceptive deficits in Parkinson's disease patients with freezing of gait. Neuroscience.

[47] Vaugoyeau M, Hakam H, Azulay JP (2011). Proprioceptive impairment and postural orientation control in Parkinson's disease. Hum Movement Sci.

[48] Brown LA, Doan JB, McKenzie NC, Cooper SA (2006). Anxiety-mediated gait adaptations reduce errors of obstacle negotiation among younger and older adults: implications for fall risk. Gait & posture.

[49] Cowie D, Limousin P, Peters A, Hariz M, Day BL (2012). Doorway-provoked freezing of gait in Parkinson's disease. Movement Disord.

